# Tumor Location in the Head/Uncinate Process and Presence of Fibrosis Impair the Adequacy of Endoscopic Ultrasound-Guided Tissue Acquisition of Solid Pancreatic Tumors

**DOI:** 10.3390/cancers14143544

**Published:** 2022-07-21

**Authors:** Thomas Togliani, Andrea Lisotti, Rosa Rinaldi, Adele Fornelli, Stefano Pilati, Nicola Passigato, Pietro Fusaroli

**Affiliations:** 1 Gastroenterology Unit, Hospital of Mantova, 46100 Mantova, Italy; stefano.pilati@asst-mantova.it; 2 Gastroenterology Unit, University Hospital Borgo Trento, 37126 Verona, Italy; nicola.passigato@aovr.veneto.it; 3 Gastroenterology Unit, Hospital of Imola, University of Bologna, 40126 Imola, Italy; lisotti.andrea@unibo.it (A.L.); pietro.fusaroli@unibo.it (P.F.); 4 Pathology Unit, Hospital of Mantova, 46100 Mantova, Italy; rosa.rinaldi@asst-mantova.it; 5 Pathology Unit, AUSL Bologna, Bellaria-Maggiore Hospital, 40133 Bologna, Italy; adele.fornelli@ausl.bologna.it

**Keywords:** endoscopic ultrasonography (EUS), pancreas, fine needle aspiration (FNA), fine needle biopsy (FNB), adequacy, fibrosis

## Abstract

**Simple Summary:**

Endoscopic ultrasound-guided tissue acquisition is the most accurate method to diagnose pancreatic tumors; nevertheless, this technique does not always bring adequate diagnostic accuracy. This study aimed to identify which factors can impair its adequacy. Pancreatic cytological and histological aspirates were retrospectively assessed according to two scores for grading the adequacy and the fibrosis of the specimens. The performance of the biopsies was lower when the tumor was located in the head/uncinate process of the pancreas, probably due to the higher fibrosis that we found in these sites. The specimens were less adequate also when <3 needle passes were performed and when the cell block was not done. We demonstrated the benefit to assess the presence of fibrosis in the specimens because it increased the risk of false negative results.

**Abstract:**

Endoscopic ultrasound-guided tissue acquisition (EUS-TA) of solid pancreatic tumors shows optimal specificity despite fair sensitivity, with an overall suboptimal diagnostic yield. We aim to quantify the adequacy and accuracy of EUS-TA and assess predictive factors for success, focusing on the presence and degree of specimen fibrosis. All consecutive EUS-TA procedures were retrieved, and the specimens were graded for sample adequacy and fibrosis. The results were evaluated according to patients’ and tumor characteristics and the EUS-TA technique. In total, 407 patients (59% male, 70 [63–77] year old) were included; sample adequacy and diagnostic accuracy were 90.2% and 94.7%, respectively. Fibrosis was significantly more represented in tumors located in the head/uncinate process (*p* = 0.001). Tumor location in the head/uncinate (OR 0.37 [0.14–0.99]), number of needle passes ≥ 3 (OR 4.53 [2.22–9.28]), and the use of cell block (OR 8.82 [3.23–23.8]) were independently related to adequacy. Severe fibrosis was independently related to false negative results (OR 8.37 [2.33–30.0]). Pancreatic tumors located in the head/uncinate process showed higher fibrosis, resulting in EUS-TA with lower sample adequacy and diagnostic accuracy. We maintain that three or more needle passes and cell block should be done to increase the diagnostic yield.

## 1. Introduction

Endoscopic ultrasound-guided tissue acquisition (EUS-TA) is the safest and most accurate technique for obtaining tissue samples of pancreatic tumors [1,2]. However, the adequacy and diagnostic accuracy of pancreatic tissue specimens still fail to reach 100%. This might even be more significant with the advent of precision medicine for pancreatic cancer, which requires representative tissue cores not only for an exact diagnosis, but also for additional molecular analyses.

Failing to obtain a diagnosis at the index procedure usually comes at relevant costs including the need for repeating procedures of EUS-TA [3] involving the cytopathologists for rapid on-site tissue evaluation [4,5,6] and ultimately delaying the proper patient treatment. Several factors have been called into question regarding either the procedure or the disease.

Several expert endosonographers have contributed to the arena of EUS-TA suggesting different methods and techniques over the years. These include increasing the number of needle passes [7] and using the fanning technique [8] along with apparently minor variations such as with/without the stylet [9] and slow pull/wet/dry/no suction [10,11]. Manufacturers from their side have produced needles of different sizes and echogenicity [12,13] with tip designs that are best suited for obtaining material either for cytology with EUS-fine needle aspiration (EUS-FNA) or for histology with EUS-fine needle biopsy (EUS-FNB), with or without side holes [14,15,16]. Some authors have also reported that a macroscopic on-site self-evaluation (MOSE) of the specimens yields good results in terms of adequacy, accuracy, and number of needle passes [17,18,19].

On the other hand, disease-related factors influencing the diagnostic accuracy of EUS-TA have been highlighted less often than the previous ones. We know that the small size and cystic nature of pancreatic lesions negatively affect the diagnostic accuracy of EUS-TA [20]. However, anatomical and histological features of the tumors have been poorly investigated in this respect. Each histological type of pancreatic tumor has a different cellular architecture, a specific microenvironment with typical vascularization, and anatomical sites of higher and lower incidence. While pancreatic ductal adenocarcinoma is characterized by a diffuse desmoplasia, consisting of a dense extracellular matrix and fibroblasts [21], acinar adenocarcinomas and neuroendocrine tumors do not show the same fibrotic stroma surrounding the neoplastic cells. It has been hypothesized that a stiff stroma [22,23] and/or pancreatic head location [24,25] can negatively affect the cellular yield of EUS-TA, but a conclusive analysis on this matter still lacks. The main aim of this study was to quantify the adequacy of pancreatic EUS-TA through a specific score while checking the influence of the characteristics of the patient, the tumor, and the technique used. The secondary aim was to quantify the fibrosis of the specimens by an original score and to assess its effect on the adequacy of pancreatic EUS-TA.

## 2. Materials and Methods

### 2.1. Study Design and Patients

We performed a retrospective analysis of a prospectively collected database to retrieve all the patients with solid pancreatic tumors who underwent EUS-TA in the period 2007–2020 at the Hospital of Mantova, Italy. The study was conducted following the principles of the Declaration of Helsinki (revision of Edinburgh, 2000).

Inclusion criteria were EUS-TA for solid pancreatic tumor, age ≥ 18 years old, and signed informed consent for EUS and tissue acquisition. Exclusion criteria were cystic pancreatic lesions (regardless of the presence of solid components), previous attempts of tissue sampling, previous endoscopic retrograde cholangio-pancreatography with or without stent placement, and missing data about pathology or follow-up.

The following variables were recorded and analyzed: patients’ gender and age, tumor size and location, needle type and size, number of needle passes, enrollment date, and preparation of the EUS-TA specimen.

### 2.2. EUS-TA

Antiplatelet and anticoagulant drugs were managed according to available guidelines. In detail, all agents except for aspirin were discontinued before EUS. All the procedures were performed by expert endosonographers (>1000 EUS procedures) under conscious sedation with meperidine and midazolam.

Equipment included linear echoendoscopes (GF UCT140 or GF UCT180, Olympus Medical System, Tokyo, Japan) attached to either the Aloka SSD-5500 (Hitachi-Aloka Medical Systems Europe, Steinhausen, Switzerland) or the EU-M60 or the EU-ME2 ultrasound processors (Olympus Medical System, Tokyo, Japan). EUS-FNA was performed with one of the following 19G, 22G, and 25G FNA needles: Cook Echotip Ultra (Cook Ltd., Limerick, Ireland), Boston-Scientific Expect (Boston Scientific Corp, Spencer, IN, USA), Olympus EZ-Shot 2 (Olympus Corp., Tokyo, Japan). EUS-FNB was performed using first-generation needles such as 22G or 25G ProCore (Cook Ltd., Limerick, Ireland), and second-generation needles such as 20G ProCore (Cook Ltd., Limerick, Ireland), 22G or 25G Acquire (Boston Scientific Corp, Spencer, IN, USA), and 22G SharkCore (Medtronic International, Tolochenaz, Switzerland). Needle and aspiration (dry suction/slow pull) were chosen according to the best available evidence in every case.

### 2.3. Material Preparation and Macroscopic Evaluation

As rapid on-site evaluation by a cytotechnician was not available, EUS-TA specimens were handled directly by the endosonographers. EUS-FNA material was slowly expelled by reinserting the stylet and a small drop of liquid was placed on each slide. The slides were smeared and macroscopically evaluated under oblique white light. The material was considered adequate when opaque thin granular or thread-like whitish material was visible and blood was either scarce or absent. Finally, the slides were fixed in a 95% alcohol solution. EUS-TA was terminated when at least five slides were deemed adequate according to the MOSE, regardless of the number of needle passes.

EUS-FNB material was expelled into a formalin vial by reinserting the stylet. The specimen was considered adequate if at least a 10 mm whitish core was floating in the liquid; otherwise, additional needle passes were repeated. The procedure was terminated when the specimen appeared sufficient according to the MOSE [26], regardless of the number of needle passes. Depending on the pathologist’s preference, the cytological specimen was also used for cell block.

### 2.4. Pathologist Work-Up

Alcohol-fixed FNA-smeared slides were stained with Papanicolau (PAP) and May Grunwald-Giemsa (MGG) solutions. Formalin-fixed FNB tissue cores were processed as histopathologic samples.

Moreover, the alcohol used for the FNA slides and the formalin used for the FNB cores were visually inspected. If cloudiness or small fragments were seen, they were centrifuged in plastic test tubes for 4 min at 1500 rpm and the supernatant was decanted. A minimum of two thin smears were prepared from the sediment according to the PAP method. PAP and Hematoxylin and Eosin (HE) staining were done. The resulting pellets were fixed and overlaid with a 10% formalin-buffered solution. Pellets were placed in a cassette, embedded in paraffin wax, and finally trimmed in 3 µm thick sections for HE staining and immunocytochemistry (cell block technique).

**Figure 1:**
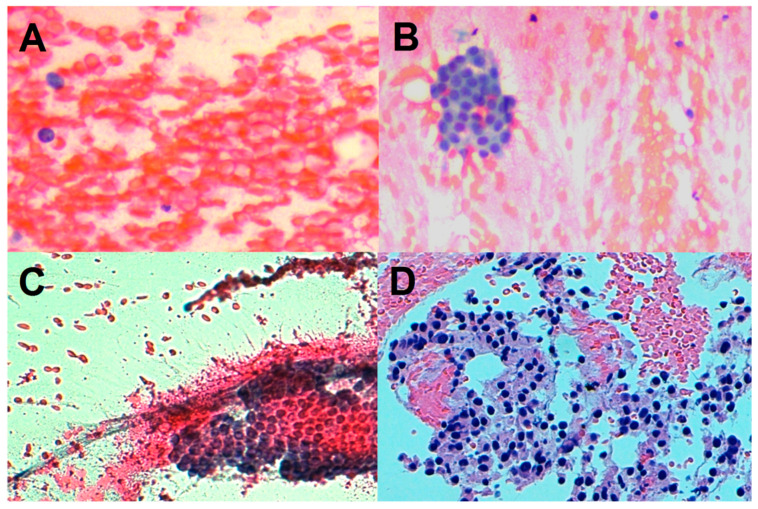
Adequacy score. (**A**): inadequate specimen (exclusively blood sample devoid of epithelial cellularity). (**B**): low cellularity (a small aggregate of epithelial cells in blood sample). (**C**): moderate cellularity (a clearly visible cellular cluster with preserved architecture, without atypia). (**D**): rich cellularity (cellular neoplastic clusters with atypical hyperchromic nuclei, punctiform necrosis, and apoptotic bodies).

*Adequacy*. In each pathology report, the proposed diagnosis together with the grade of specimen adequacy and tumor fibrosis were described. The adequacy was evaluated by assessing the presence of the neoplastic cellular component in the cyto-histological preparations, initially with low-power magnification (6.3× and 10×), then with high-power magnification (20× and 40×). Adequacy was graded according to the following classification: 0—inadequate (entirely or mainly represented by blood or contaminating gastric or duodenal mucosal flaps, with less than 100 neoplastic cells); 1—poor cellularity (just above the threshold of 100 pancreatic cells, visible only with high-power magnification); 2—moderate cellularity (cellular clusters visible in some low-power fields and many high-power fields); 3—rich cellularity (cellular clusters visible in many low-power fields and all high-power fields) [27] (Figure 1).

*Fibrosis*. Since no standardized fibrosis score in EUS-TA pancreatic specimens was available, the grade of fibrosis was quantified according to a scoring system derived from breast fine needle biopsy [28].

**Figure 2:**
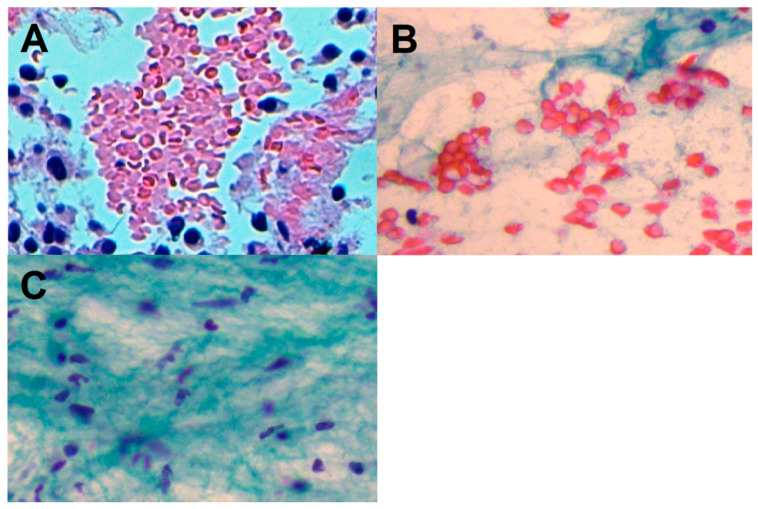
Fibrosis score. (**A**): fibrosis grade 0 (cellular neoplastic clusters; total absence of fibrous tissue). (**B**): fibrosis grade 1 (wispy, loose density fibrous tissue with admixture of moderate cellular epithelial component). (**C**): fibrosis grade 2 (clearly visible loose fibrous tissue fragment; absence of epithelial component).

Fibrosis was graded according to the following classification: grade 0 (total absence of fibrous tissue, desmoplastic stroma, or wispy collagen fibers in cytological smears or cell block; only the neoplastic cells are well represented); grade 1 (presence of 1–2 wispy collagen fibers at 40× magnification and/or presence of loose to moderate density fibrous tissue or desmoplastic stroma with an admixture of moderate cellular epithelial neoplastic component); grade 2 (presence of ≥3 wispy collagen fibers at 40× magnification and/or presence of dense fibrous tissue or well-evident desmoplastic stroma, with an admixture of less represented cellular epithelial neoplastic component) (Figure 2).

By definition, loose density stroma is characterized by a loose fibroblastic myxoid stroma and occasional short wispy collagen fibers. Moderate density stroma is composed of interrupted bands of keloid-like collagen (collagen with brightly eosinophilic hyalinization, similarly to keloid) without myxoid changes. High-density stroma presents mature collagen fibers, i.e., fine elongated collagen fibers densely packed into multilayers with intense staining lacking keloid-like collagen bands.

*Final diagnosis—gold standard.* The gold standard for the diagnosis was the pathological evaluation on the surgical specimens, when available. If surgery was not indicated, the data from EUS-TA results together with radiological and clinical follow-up were integrated.

In the case of non-diagnostic or inconclusive EUS-TA, patients were strictly followed-up; the decision of repeating EUS-TA or performing other diagnostic and/or therapeutic interventions was based on the multidisciplinary evaluation. True negative EUS-TA was considered if the patients did not show any clinical, radiological, or histological signs of malignancy after at least one year of follow-up.

### 2.5. Statistical Analysis

Categorical variables were reported as number (no.) and percentage (%), while continuous variables as means ± standard deviation or median [interquartile range] when appropriate. Univariate and multivariate analyses were used to identify variables related to specimen adequacy, good adequacy (score 2 or 3), and risk factors for false negative EUS-TA results; odds ratio (OR) and 95% confidence interval (95% CI) were reported. Statistical analysis was performed using MedCalc^®^ Statistical Software version 19.5.2 (MedCalc Software Ltd., Ostend, Belgium; https://www.medcalc.org; accessed on 1 March 2020).

## 3. Results

### 3.1. Study Population

Four hundred and seven patients were included. In all cases, the EUS-guided tissue acquisition was technically feasible. Baseline characteristics are shown in Table 1.

In detail, 240 males (59.0%) with median age of 70 [63–77] years old entered the study. In total, 287 tumors (70.5%) were located in the head/uncinate process, while the remaining 120 (29.5%) were in the neck, body, and tail of the pancreas. Median tumor size was 31 [24–40] mm. EUS-TA was performed in 316 (77.6%) cases using EUS-FNA needles; among the remaining 91 cases (22.4%), 23 were conducted using 1first-generation FNB needles and 68 with second-generation needles. A median of 3 needle passes [1–4] was performed. In 316 cases (77.6%), EUS-TA specimens were placed over slides, while in the remaining 91 (22.4%) cases they were placed inside formalin vials. In 182 (44.7%) cases, cell block was performed. No adverse event was observed. MOSE was satisfactory in all cases.

Pathology reports are described in Table 2.

In detail, a conclusive diagnosis was possible in 319 cases, while in 48 cases the pathological report was just negative for malignant cells, without any further specification. Finally, in 40 cases, the specimen was inadequate; of these, 20 were diagnosed with a pancreatic malignancy by further investigations and 20 were lost at follow up.

### 3.2. Sample Adequacy

EUS-TA tissue sample was considered adequate in 367 (90.2%) cases. On univariate analysis, patients’ age (OR 1.02 [1.00–1.05]; *p* = 0.05), tumor size (OR 1.03 [1.01–1.06]; *p* = 0.04), tumor located in head/uncinate process (OR 0.31 [0.12–0.82]; *p* = 0.02), the performance of ≥3 needle passes (OR 3.01 [1.54–5.88]; *p* = 0.001), and cell block (6.52 [2.50–17.0]; *p* = 0.001) were related to adequacy. Multivariate analysis identified tumor location (OR 0.37 [0.14–0.99]; *p* = 0.05), needle passes ≥3 (OR 3.01 [1.54–5.88]; *p* < 0.001), and cell block (OR 6.52 [2.50–17.0]; *p*< 0.001) independently related to sample adequacy (Table 3).

Sub-group analyses evaluating sample adequacy according to tumor location are shown in Table 4 and Table 5.

In detail, age (OR 1.07 [1.03–1.11; *p* = 0.002), use of second-generation FNB needle (OR 2.25 [1.05–3.22]; *p* = 0.03), and fibrosis grade ≥1 (OR 0.30 [0.15–0.38]; *p* = 0.01) were independently related to sample adequacy for tumors located in the head/uncinate process. On the other hand, male gender (OR 0.11 [0.01–0.93]; *p* = 0.04) and tumor size (OR 1.10 [1.03–1.18]; *p* = 0.006) were independently related to sample adequacy for tumors located in the neck, body, and tail of the pancreas.

### 3.3. Adequacy Score 2 or 3

According to the proposed score, EUS-TA samples presented a score of 2 or 3 in 139 (34.2%) and 180 (44.2%) cases, respectively.

Univariate and multivariate analyses of factors related to good sample adequacy (score 2 or 3) are shown in Table 6.

Patients’ age (OR 1.02 [1.00–1.04]; *p* = 0.03), study period (2015–2020) (OR 2.00 [1.24–3.23]; *p* = 0.004), tumor size (OR 1.03 [1.01–1.05]; *p* = 0.003), location (head/uncinate process) (OR 0.42 [0.23–0.77]; *p* = 0.005), needle passes ≥ 3 (OR 2.26 [1.40–3.65]; *p* = 0.001), cell block (OR 1.76 [1.08–2.88]; *p* = 0.02), fibrosis grade ≥ 1 (OR 0.28 [0.17–0.47]; *p* < 0.001), and fibrosis grade = 2 (OR 0.03 [0.01–0.06]; *p* < 0.001) were related to good sample adequacy. On multivariate analysis, study period (2015–2020) (OR 2.07 [1.12–3.82]; *p* = 0.02), tumor size (OR 1.04 [1.01–1.06]; *p* = 0.009), and fibrosis grade = 2 (OR 0.03 [0.01–0.06]; *p* < 0.001) were independently related to sample adequacy.

### 3.4. Tumor Fibrosis

Among the entire study population (Table 1), 217 (53.3%) tumors presented no fibrosis (grade 0), 127 (31.2%) presented low-to-moderate fibrosis (grade 1) and 18 (4.4%) presented severe fibrosis (grade 2). The 40 inadequate cases were excluded from the assessment of the inflammatory microenvironment. Among the remaining adequate 367 cases, fibrosis was evaluable in 362 (98.6%) cases.

Fibrosis was significantly higher for tumors located in the head/uncinate process, compared to the neck, body, and tail of the pancreas (Table 7).

The lack of fibrosis was more frequently observed in the neck, body, and tail (71.1% vs. 54.8%), while low-to-moderate fibrosis (24.6% vs. 39.9%) and severe fibrosis (5.2% vs. 4.4%) were more frequently displayed in the head/uncinate process (*p* = 0.001). A lower fibrosis grade was also observed in EUS-TA with good adequacy, compared to samples with low adequacy (Table 7).

### 3.5. Diagnostic Performance

Overall sensitivity was 94.4% [91.3–96.6%] and specificity was 97.3% [85.8–99.9%]. The positive likelihood ratio was 34.9 [5.1–241.5], and the negative likelihood ratio was 0.06 [0.04–0.09]. The positive and negative predictive values were 99.7% [97.7–99.9%] and 66.7 [56.0–75.9%], respectively. Overall diagnostic accuracy was 94.7% [91.8–96.8%]. In detail, 18 false negative cases were identified during follow-up and 1 false positive case was identified on the surgical specimen, after pancreatectomy. Table 8 shows variables related to false negative EUS-TA results.

On univariate analysis, needle design (FNA vs. FNB) (OR 2.93 [1.11–7.72]; *p* = 0.03) and severe fibrosis (OR 8.37 [2.33–30.0]; *p*= 0.001) were related to false negative results. On multivariate analysis, severe fibrosis was the only risk factor for false negative EUS-TA results (OR 8.37 [2.33–30.0]; *p*= 0.001).

The subgroup of 48 patients that had a non-malignant biopsy showed peculiar characteristics: 35 (72.9%) of the lesions were located in the head/uncinate process; the mean adequacy rate was low (21 with adequacy score 1, 26 with score 2, and just 1 with score 3); a whatever degree of fibrosis (grade 1 or 2) was found in 15 (34.1%) out of 44 cases (in 4 cases the fibrosis was not evaluable).

## 4. Discussion

In this large single-center study, EUS-TA of solid pancreatic tumor emerged as a technically feasible and safe procedure, with overall adequacy of about 90%, including small lesions. For the first time, a four-level score measuring the degree of adequacy was applied to better analyze the variables influencing the performance of EUS-TA.

Our data showed that either a minimum of three needle passes or cell block were the most important technical factors increasing the adequacy of the aspirate, regardless of the type of the needle used. In our series, this combination provided a 98% adequacy [29].

We showed that the adequacy of EUS-TA was influenced by pancreatic tumor location. The procedures via a trans-duodenal route (towards the head/uncinate process) as compared to those via a trans-gastric route (towards the neck, body, and tail) had either an overall lower adequacy rate or a worse adequacy score. As the difference between the two puncture locations did not depend on the technical success of the procedure, we looked for other explanations.

Since other authors previously postulated that the stiff stroma of pancreatic tumors could decrease the performance of EUS-TA, we tried to quantify the fibrosis of the EUS-TA specimens to assess its possible relationship with the adequacy of the procedure. As it is hard to quantify precisely the fibrosis of a fine needle sample, we adapted a three-level grade from breast tumors to obtain an approximate quantification of the collagen fibers and the desmoplastic stroma. We then found two interesting facts. First, grades 1 and 2 of fibrosis were more frequent in the head/uncinate process (47.3% of cases) than in the neck, body, and tail (31.4% of cases), independently from the histotype of the lesion. Second, the presence of fibrosis, probably encasing the neoplastic cells in a dense, stiff matrix rich in fibroblasts, impaired not only the accuracy, but also the adequacy of EUS-TA samples.

These results had practical consequences as the head/uncinate process were either the most prevalent sites of tumors or the regions where the majority of poorly adequate and non-diagnostic biopsies occurred. Thus, we assume that to biopsy a focal lesion of the head/uncinate process in the absence of ROSE, it is extremely important to optimize our performance by performing at least three needle passes and the cell block. Additionally, we speculate that in the case of solid tumors with severe fibrosis, the use of ancillary techniques, such as contrast-enhanced harmonic guided tissue acquisition, could be used to increase the EUS-TA diagnostic yield [30].

Another meaningful information occurred when the specimen of a pancreatic focal lesion resulted in a non-malignant finding. These tumors were prevalently located in the head/uncinate process and showed a high fibrotic pattern and a low mean adequacy of EUS-TA. Moreover, these patients had a poor prognosis at follow up, showing all the same characteristics of pancreatic ductal adenocarcinoma. Thus, when a report comes back from the cytopathologist generically labeled as non-malignant, our suggestion is to consider that the degree of fibrosis is associated with a low accuracy of EUS-TA in these cases.

We acknowledge several limitations of our study. First, the two proposed scoring systems for adequacy and fibrosis have not been validated for EUS-TA. Therefore, our results should be verified by further investigations. Second, the long study period may have induced a temporal bias; however, we analyzed our results also according to the study period when EUS-TA was performed and found no significant difference in terms of adequacy and accuracy.

## 5. Conclusions

The adequacy of pancreatic EUS-TA was directly correlated with the performance of three or more needle passes and cell block but was negatively affected by the location of the lesion in the head/uncinate process. Presence and grading of tissue fibrosis were higher in lesions located in the head/uncinate process and seemed to be responsible for the negative impact on sample adequacy. We proposed two scores to quantify the degree of adequacy and grade of fibrosis of EUS-TA specimens; these results could stratify the risk of a false negative diagnosis. Further larger studies are recommended to confirm the low adequacy rate and the high prevalence of fibrosis in the head/uncinate process and to establish the applicability of our scores to EUS-TA.

## Figures and Tables

**Table 1:** Baseline characteristics of the study population.

Characteristic	Total (No. 407)
*Demographic*	
Gender (male), *no.* (*%*)	240 (59.0%)
Age (years), *median* [*IQR*]	70 [63–77]
Study period 2007–2014	181 (44.5%)
Study period 2015–2020	226 (55.5%)
*Pancreatic tumor*	
Size (mm), *median* [*IQR*]	31 [24–40]
Head, *no.* (*%*)	255 (62.7%)
Uncinate process, *no.* (*%*)	32 (7.9%)
Neck, *no.* (*%*)	32 (7.9%)
Body, *no.* (*%*)	52 (12.8%)
Tail, *no.* (*%*)	36 (8.8%)
*Needle design*	
FNA-needle, *no.* (*%*)	316 (77.6%)
FNB-needle, *no.* (*%*)	91 (22.4%)
1st generation FNB-needle, *no.*	23
2nd generation FNB-needle, *no.*	68
*Needle size*	
19-gauge needle, *no.* (*%*)	5 (1.2%)
20-gauge needle, *no.* (*%*)	63 (15.5%)
22-gauge needle, *no.* (*%*)	191 (46.9%)
25-gauge needle, *no.* (*%*)	148 (36.4%)
*Needle passes*	
Number of needle passes, *median* [*IQR*]	
No. 1, *no.* (*%*)	50 (12.3%)
No. 2, *no.* (*%*)	96 (23.6%)
No. 3, *no.* (*%*)	132 (32.4%)
No. 4, *no.* (*%*)	68 (16.7%)
No. 5, *no.* (*%*)	61 (15.0%)
*EUS-TA specimen*	
Slides, *no.* (*%*)	316 (77.6%)
Formalin vials, *no.* (*%*)	91 (22.4%)
Cell-block, *no.* (*%*)	182 (44.7%)
*Pathology–adequacy*	
Overall adequacy, *no.* (*%*)	367 (90.2%)
Adequacy–Score 0, *no.* (*%*)	40 (9.8%)
Adequacy–Score 1, *no.* (*%*)	48 (11.8%)
Adequacy–Score 2, *no.* (*%*)	139 (34.2%)
Adequacy–Score 3, *no.* (*%*)	180 (44.2%)
*Pathology–fibrosis*	
Fibrosis–not evaluable, *no.* (*%*)	45 (11.1%)
Fibrosis–Score 0, *no.* (*%*)	217 (53.3%)
Fibrosis–Score 1, *no.* (*%*)	127 (31.2%)
Fibrosis–Score 2, *no.* (*%*)	18 (4.4%)
Accuracy, *no.* (*%*)	339 (94.7%) *

Abbreviations: EUS—endoscopic ultrasound; IQR—interquartile range; FNA—fine needle aspiration; FNB—fine needle biopsy; EUS-TA—EUS-guided tissue acquisition. * Accuracy calculated as the proportion of accurate case among patients with final diagnosis (see Section 2).

**Table 2:** Pathological diagnosis of EUS-tissue acquisition.

EUS-TA Pathological Diagnosis	Total (No. 407)
Adenocarcinoma, *no.* (*%*)	268 (65.9%)
Ductal, *no.*	254
Mucinous, *no.*	6
Mixed adeno-squamous, *no.*	3
Acinar, *no.*	2
Hepatoid, *no.*	1
Micro-glandular, *no.*	1
Pleomorphic, *no.*	1
Cholangiocarcinoma, *no.* (*%*)	1 (0.2%)
Well-differentiated neuroendocrine neoplasm, *no.* (*%*)	15 (3.7%)
Neuroendocrine carcinoma, *no.* (*%*)	3 (0.7%)
Chronic pancreatitis, *no.* (*%*)	10 (2.5%)
Autoimmune pancreatitis, *no.* (*%*)	2 (0.5%)
Metastasis, *no.* (*%*)	10 (2.5%)
Renal cancer, *no.*	7
Lung cancer, *no.*	2
Lung neuroendocrine carcinoma, *no.*	1
Benign lymphoid tissue, *no.* (*%*)	10 (2.5%)
Benign pancreatic cells with no atypia, *no.* (*%*)	48 (11.8%)
Non-diagnostic, *no.* (*%*)	40 (9.8%)

**Table 3:** Variables related to EUS-guided tissue acquisition specimen adequacy.

Adequacy of EUS-TA				
	Univariate Analysis (OR [95% C.I.)	*p*	Multivariate Analysis (OR [95% C.I.)	*p*
Gender (male)	ns	ns	---	---
Age	1.02 [1.00–1.05]	0.05	ns	ns
Study period 2015–2020	ns	ns	---	---
Size	1.03 [1.01–1.06]	0.04	ns	ns
Location (head/uncinate process)	0.31 [0.12–0.82]	0.02	0.37 [0.14–0.99]	0.05
FNA needle	ns	ns	---	---
FNB needle	ns	ns	---	---
Second-gen. FNB needle	ns	ns	---	---
19-gauge needle	ns	ns	---	---
20-gauge needle	ns	ns	---	---
22-gauge needle	ns	ns	---	---
25-gauge needle	ns	ns	---	---
22G or 25G needle	ns	ns	---	---
Needle passes ≥ 3	3.01 [1.54–5.88]	0.001	4.53 [2.22–9.28]	<0.001
Slides	ns	ns	---	---
Cell block	6.52 [2.50–17.0]	0.001	8.82 [3.26–23.8]	<0.001
Score fibrosis 1 or 2	ns	ns	---	---

Abbreviations: OR—Odds ratio; 95% C.I.—95% confidence interval; ns—not statistically significant; EUS—endoscopic ultrasound; FNA—fine needle aspiration; FNB—fine needle biopsy; EUS-TA—EUS-guided tissue acquisition.

**Table 4:** Variables related to EUS-guided tissue acquisition specimen adequacy for solid pancreatic tumors located in the head/uncinate process.

Adequacy of EUS-TA				
	Univariate Analysis (OR [95% C.I.)	*p*	Multivariate Analysis (OR [95% C.I.)	*p*
Gender (male)	ns	ns	---	---
Age	1.02 [1.00–1.06]	0.05	1.07 [1.03–1.11]	0.002
Study period 2015–2020	2.05 [1.01–4.25]	0.01	ns	ns
Size	ns	ns	---	---
FNA needle	ns	ns	---	---
FNB needle	ns	ns	---	---
Second-gen. FNB needle	4.26 [1.10–7.04]	0.03	2.25 [1.05–3.22]	0.03
19-gauge needle	ns	ns	---	---
20-gauge needle	ns	ns	---	---
22-gauge needle	ns	ns	---	---
25-gauge needle	ns	ns	---	---
22G or 25G needle	ns	ns	---	---
Needle passes ≥ 3	ns	ns	---	---
Slides	ns	ns	---	---
Cell block	ns	ns	---	---
Score fibrosis 1 or 2	0.25 [0.12–0.62]	0.04	0.30 [0.15–0.38]	0.01

Abbreviations: OR—Odds ratio; 95% C.I.—95% confidence interval; ns—not statistically significant; EUS—endoscopic ultrasound; FNA—fine needle aspiration; FNB—fine needle biopsy; EUS-TA—EUS-guided tissue acquisition.

**Table 5:** Variables related to EUS-guided tissue acquisition specimen adequacy for solid pancreatic tumors located in the neck, body, and tail.

Adequacy of EUS-TA				
	Univariate Analysis (OR [95% C.I.)	*p*	Multivariate Analysis (OR [95% C.I.)	*p*
Gender (male)	0.12 [0.02–0.97]	0.05	0.11 [0.01–0.93]	0.04
Age	ns	ns	---	---
Study period 2015–2020	ns	ns	---	---
Size	1.10 [1.03–1.18]	0.005	1.10 [1.03–1.18]	0.006
FNA needle	ns	ns	---	---
FNB needle	ns	ns	---	---
Second-gen. FNB needle	ns	ns	---	---
19-gauge needle	ns	ns	---	---
20-gauge needle	ns	ns	---	---
22-gauge needle	ns	ns	---	---
25-gauge needle	ns	ns	---	---
22G or 25G needle	ns	ns	---	---
Needle passes ≥ 3	ns	ns	---	---
Slides	ns	ns	---	---
Cell block	ns	ns	---	---
Score fibrosis 1 or 2	ns	ns	---	---

Abbreviations: OR—Odds ratio; 95% C.I.—95% confidence interval; ns—not statistically significant; EUS—endoscopic ultrasound; FNA—fine needle aspiration; FNB—fine needle biopsy; EUS-TA—EUS-guided tissue acquisition.

**Table 6:** Variables related to good adequacy (score of 2 or 3) of EUS-guided tissue acquisition specimen.

Adequacy of EUS-TA				
	Univariate Analysis (OR [95% C.I.)	*p*	Multivariate Analysis (OR [95% C.I.)	*p*
Gender (male)	ns	ns	---	---
Age	1.02 [1.00–1.04]	0.03	ns	ns
Study period 2015–2020	2.00 [1.24–3.23]	0.004	2.07 [1.12–3.82]	0.02
Size	1.03 [1.01–1.05]	0.003	1.04 [1.01–1.06]	0.009
Location (head/uncinate process)	0.42 [0.23–0.77]	0.005	ns	ns
FNA needle	ns	ns	---	---
FNB needle	ns	ns	---	---
Second-gen. FNB needle	ns	ns	---	---
19-gauge needle	ns	ns	---	---
20-gauge needle	ns	ns	---	---
22-gauge needle	ns	ns	---	---
25-gauge needle	ns	ns	---	---
22G or 25G needle	ns	ns	---	---
Needle passes ≥ 3	2.26 [1.40–3.65]	0.001	ns	ns
Slides	ns	ns	---	---
Cell block	1.76 [1.08–2.88]	0.02	ns	ns
Score fibrosis 1 or 2	0.28 [0.17–0.47]	<0.001	ns	ns
Score fibrosis 2	0.03 [0.01–0.06]	<0.001	0.03 [0.01–0.06]	<0.001

Abbreviations: OR—Odd ratio; 95% C.I.—95% confidence interval; ns—not statistically significant; EUS—endoscopic ultrasound; FNA—fine needle aspiration; FNB—fine needle biopsy; EUS-TA—EUS-guided tissue acquisition.

**Table 7:** Fibrosis distribution according to tumor location (head/uncinate process vs. neck, body, and tail) and according to specimen adequacy.

	Score Fibrosis = 0 (No. 217)	Score Fibrosis = 1 (No. 127)	Score Fibrosis = 2 (No. 145)	*p* *
Head/uncinate process (no. 248)	136 (54.8%)	99 (39.9%)	13 (5.2%)	
Neck, body, and tail (no. 114)	81 (71.1%)	28 (24.6%)	5 (4.4%)	0.001
Score adequacy = 1 (no. 45)	26 (57.8%)	11 (24.4%)	8 (17.8%)	
Score adequacy = 2 (no. 137)	81 (59.1%)	50 (36.5%)	6 (4.4%)	
Score adequacy = 3 (no. 180)	110 (61.1%)	66 (36.7%)	4 (2.2%)	0.001

* *p* values calculated used Chi-square test.

**Table 8:** Variables related to false negative EUS-guided tissue acquisition results.

Adequacy of EUS-TA				
	Univariate Analysis (OR [95% C.I.)	*p*	Multivariate Analysis (OR [95% C.I.)	*p*
Gender (male)	ns	ns	---	---
Age	ns	ns	---	---
Study period 2015–2020	ns	ns	---	---
Size	ns	ns	---	---
FNA needle	2.93 [1.11–7.72]	0.03	ns	ns
FNB needle	0.34 [0.13–0.90]	0.03	ns	ns
Second-gen. FNB needle	ns	ns	---	---
19-gauge needle	ns	ns	---	---
20-gauge needle	ns	ns	---	---
22-gauge needle	ns	ns	---	---
25-gauge needle	ns	ns	---	---
22G or 25G needle	ns	ns	---	---
Needle passes ≥ 3	ns	ns	---	---
Slides	ns	ns	---	---
Cell block	ns	ns	---	---
Score fibrosis 1 or 2	ns	ns	---	---
Score fibrosis 2	8.37 [2.33–30.0]	0.001	8.37 [2.33–30.0]	0.001

Abbreviations: OR—Odd ratio; 95% C.I.—95% confidence interval; ns—not statistically significant; EUS—endoscopic ultrasound; FNA—fine needle aspiration; FNB—fine needle biopsy; EUS-TA—EUS-guided tissue acquisition.
